# A New Role for TIMP-1 in Modulating Neurite Outgrowth and Morphology of Cortical Neurons

**DOI:** 10.1371/journal.pone.0008289

**Published:** 2009-12-14

**Authors:** Adlane Ould-yahoui, Evelyne Tremblay, Oualid Sbai, Lotfi Ferhat, Anne Bernard, Eliane Charrat, Yatma Gueye, Ngee Han Lim, Keith Brew, Jean-Jacques Risso, Vincent Dive, Michel Khrestchatisky, Santiago Rivera

**Affiliations:** 1 Neurobiologie des Interactions Cellulaires et Neurophysiopathologie (NICN), UMR 6184, Centre National de la Recherche Scientifique (CNRS) - Université de la Méditerranée, Marseille, France; 2 Kennedy Institute of Rheumatology Division, Imperial College of London, London, United Kingdom; 3 Department of Biomedical Sciences, Florida Atlantic University, Boca Raton, Florida, United States of America; 4 Département de Recherche Marine et Subaquatique, IMNSSA, UMR MD2 PPCOE, Université de la Méditerranée, Toulon Armées, France; 5 Département d'Ingénierie et d'Etudes des Protéines (DIEP), Commissariat à l'Energie Atomique (CEA), Gif-sur-Yvette, France; Institut de la Vision, France

## Abstract

**Background:**

Tissue inhibitor of metalloproteinases-1 (TIMP-1) displays pleiotropic activities, both dependent and independent of its inhibitory activity on matrix metalloproteinases (MMPs). In the central nervous system (CNS), TIMP-1 is strongly upregulated in reactive astrocytes and cortical neurons following excitotoxic/inflammatory stimuli, but no information exists on its effects on growth and morphology of cortical neurons.

**Principal Findings:**

We found that 24 h incubation with recombinant TIMP-1 induced a 35% reduction in neurite length and significantly increased growth cones size and the number of F-actin rich microprocesses. TIMP-1 mediated reduction in neurite length affected both dendrites and axons after 48 h treatment. The effects on neurite length and morphology were not elicited by a mutated form of TIMP-1 inactive against MMP-1, -2 and -3, and still inhibitory for MMP-9, but were mimicked by a broad spectrum MMP inhibitor. MMP-9 was poorly expressed in developing cortical neurons, unlike MMP-2 which was present in growth cones and whose selective inhibition caused neurite length reductions similar to those induced by TIMP-1. Moreover, TIMP-1 mediated changes in cytoskeleton reorganisation were not accompanied by modifications in the expression levels of actin, βIII-tubulin, or microtubule assembly regulatory protein MAP2c. Transfection-mediated overexpression of TIMP-1 dramatically reduced neuritic arbour extension in the absence of detectable levels of released extracellular TIMP-1.

**Conclusions:**

Altogether, TIMP-1 emerges as a modulator of neuronal outgrowth and morphology in a paracrine and autrocrine manner through the inhibition, at least in part, of MMP-2 and not MMP-9. These findings may help us understand the role of the MMP/TIMP system in post-lesion pre-scarring conditions.

## Introduction

TIMP-1 is the founding member of the MMP inhibitor family encompassing four proteins with pleiotropic actions (TIMP-1-4). In addition to inhibitory activity on proteinases hosted in the N-terminal domain of the molecule, the TIMPs display MMP-independent activities that regulate the behaviour of a wide range of cell types [1]. The poorly known interactions with non metalloproteinases involving the C-terminal domain of TIMPs may contribute to their pleiotropic effects [2]. In the CNS, TIMP-1 was first characterised as a candidate plasticity gene induced by seizures and by stimuli leading to long term potentiation (LTP) [3], a form of synaptic plasticity considered as a cellular substrate of learning and memory. In developmental/physiological conditions, TIMP-1 expression is very low and basically restricted to the hippocampus and the cerebellum during the first postnatal weeks, at a time of intense dendro-axonic remodelling [4], [5]. In clear contrast with physiological conditions, in the pathological brain TIMP-1 expression is dramatically induced in a neuronal activity-dependent manner in cortical and hippocampal neurons resistant to excitotoxicity [4]. Despite prominent neuronal expression, inflammation-driven TIMP-1 production by reactive astrocytes prevails as the main source of the inhibitor in the pre-scarring zones of lesion following seizures [4], cerebral ischemia [6], experimental autoimmune encephalomyelitis [7], intracranial injury [8], and viral infection [9]. Accordingly, TIMP-1 is upregulated in models of scarring non-regenerating optic nerve transection and downregulated in nonscarring regenerating conditions [10]. *In vitro*, proinflammatory cytokines such as TNF-α or IL-1 upregulate TIMP-1 expression in astrocytes [11], [12] which stimulates astrocyte proliferation [13].

The upregulation of TIMP-1 in the pathological nervous system is generally concomitant with the upregulation of some of its main targets, MMP-9 and MMP-2 [14], [15] and pinpoints the importance of controled proteolytic balance in the physiopathological outcome. In support of this idea, we have recently demonstrated that, unlike wild type (WT) mice, TIMP-1 deficient mice do not exhibit MMP-2 and MMP-9 upregulation after excitotoxic seizures, and this correlates with the absence of mossy fibre sprouting in the hippocampus of mutant mice [16]. The idea that these MMPs contribute to neurite outgrowth in the CNS finds support in previous studies implicating MMP-9 in neurite extension of cerebellar neurons [17] and in recent data indicating that MMP-3 and MMP-2 specifically promote axonal [18] and dendritic [19] growth in cortical neurons, respectively. Moreover, MMP-2 has been found to stimulate neurite outgrowth of dorsal root ganglia (DRG) neurons in the peripheral nervous system [20]. Although these data suggest the involvement of TIMP-1 in post-lesion structural changes of cortical and hippocampal neurons [21], [22], we still lack direct evidence that TIMP-1, whether it is produced by neurons or by surrounding glial cells, influences such changes. A way of addressing this question is to investigate the impact of physiopathological concentrations of TIMP-1 in neuronal morphology. We have combined a systematic morphometric analysis of cultured cortical neurons with the use of pseudophosphinic synthetic MMP inhibitors [16], [23], mouse recombinant full length TIMP-1, and recombinant WT and mutated inactive forms of the TIMP-1 N-terminal domain [24] previously used to demonstrate the determinant role of the N-terminal domain in the inhibition of apoptosis in non neural cells [25]. Overall, TIMP-1 emerges as a modulator of neuronal outgrowth and morphology through paracrine and autrocrine actions and involves, at least in part the inhibition of MMP-2 but not MMP-9. These findings may be important to understand the role of the MMP/TIMP system in post-lesion pre-scarring conditions.

## Materials and Methods

### Neuronal Cultures and Treatments

Primary cultures of cortical neurons were prepared from CD1 mice embryos according to the guidelines of the Ethics Committee of the Medical Faculty of Marseilles and conform to National and European regulations (EU directive N° 86/609). Pregnant females were deeply anesthetised with halothane (Nicholas Piramal Limited, London, UK) and their placenta with embryos at E17–18 were quickly removed and placed into cold HBSS containing 0.5% glucose (both from Invitrogen, Carlsbad, CA, USA). Usually, 4 embryos were used for each preparation. They were decapitated and the cerebral cortices dissected, pooled and enzymatically dissociated for 10 min at 37°C in HBSS containing 0.1% trypsine and 10 µg/ml DNase I (Sigma-Aldrich, Saint Louis, MO, USA). The reaction was stopped by replacing the trypsin medium with HBSS containing 10% foetal calf serum (Invitrogen). Further mechanical dissociation was carried out in HBSS containing 5 µg/ml of DNase by trituration through a Pasteur pipette. After centrifugation at 1250 rpm during 5 min at room temperature, the pelleted cells were resuspended in the plating medium containing MEM, 0.6% glucose, 1 mM sodium pyruvate, 5 U/ml penicillin/streptomycin and 10% foetal calf serum (all from Invitrogen). The cells were plated at a seeding density of 1.5 10^5^ cells per well onto 12 mm diameter glass coverslips precoated with 1 mg/ml poly-D-lysine (Sigma-Aldrich) in borate buffer pH 8.5, and were grown at 37°C in a humidified chamber containing 5% C0_2_. After 90 min the plating medium was aspirated and replaced by a serum free defined medium consisting in Neurobasal, with 2% B27 supplement, 5 U/ml penicillin/streptomycin, 2.5 mM glutamine (all from Invitrogen) and 25 µM glutamate (Sigma-Aldrich). The following MMP inhibitors were added to cultures in serum free media: mouse recombinant TIMP-1 (mrTIMP-1, R&D System, Minneapolis, MN, USA) in (50 mM Tris, 10 mM CaCl_2_, 150 mM NaCl, 0.05% Brij-35, pH 7.5) (TCNB); human recombinant truncated N-terminal form of TIMP-1 (NT1) with similar *K_i_* values (0.2–0.4 nM) for MMP-1, MMP-2, MMP-3 and MMP-9 and 146 nM for MT1-MMP; and mutated inactive forms of TIMP-1 (ΔNT1), in which the substitution of threonin in position 2 by glycine increases the *K_i_* value for MMP-1, MMP-2, MMP-3 and MT1-MMP by at least two to three orders of magnitude [24]. ΔNT1 preserves a good inhibitory activity for MMP-9 and exhibits a >2500-fold higher affinity for MMP-9 relative to MMP-2 [26]; MMP selective pseudophosphinic RXPO3R inhibitor that does not inhibit adamalysins [23], with *Ki* for MMP-2, MMP-9 and MT1-MMP of 55, 41 and 91 nM, respectively [27]; selective MMP-2 inhibitor (MMP-2 inhibitor III, Calbiochem, La Jolla, CA, USA), which exhibits good selectivity for MMP-2 (*Ki* 12 nM) when compared with MMP-9 and MMP-3 (*Ki* 200 and 4500 nM, respectively). Synthetic inhibitors were dissolved in DMSO 0.04% final concentration. Controls were exposed to the same concentration of DMSO.

### Primary Astrocyte Cultures

Astrocytes were obtained from 2 days old CD1 mice brains as described previously [28]. After removal of the meninges, the brains were dissociated into a single-cell suspension by trypsinisation and mechanical disruption. The cells were seeded on onto 12 mm diameter glass coverslips and grown at 37°C in a 5% CO_2_ humidified atmosphere in Dulbecco's modified Eagle medium (DMEM) containing 10% foetal calf serum, 2 mM L-glutamine, penicillin (100 U/ml), and streptomycin (100 µg/ml) (all from Invitrogen). The medium was replaced every 3 days for 2 weeks until reaching cell confluence and then replaced by a serum free medium containing DMEM, L-glutamine, penicillin, and streptomycin for 24 h.

### Culture of Neuroblastoma Cells

Mouse neuroblastoma cells (N2a, also known as Neuro 2a, ATCC, CCL-131™) were grown at 37°C in a humidified atmosphere containing 5% CO_2_ in DMEM supplemented with 10% foetal bovine serum. Cells were grown to semi-confluence for 24 h before transfection (see below).

### Reverse Transcription-Polymerase Chain Reaction (RT-PCR)

Total RNA was isolated after lysis of cultured neurons, N2a cells or astrocytes. Following OD assessment and gel analysis, 500 ng total RNA was reverse transcribed. Traces of genomic DNA were eliminated by a DNase I treatment for 15 min at 37°C. cDNAs were used for amplification of TIMP-1, MMP-2, MMP-9, β-actin and GAPDH, with the following cycling profile: 40 sec at 94°C, 40 sec at 58°C, 40 sec at 72°C for 28 to 35 cycles. The PCR products were analysed in 2% ethidium bromide stained agarose gels. The forward (F) and reverse (R) primer sequences for the different PCR products and their size in base pairs (bp) were as follows:

Timp-1 F: GGCATCCTCTTGTTGCTATCACT,

Timp-1 R: CTTATGACCAGGTCCGAGTTGC (101 bp);

MMP-2 F: AGCGTGAAGTTTGGAAGCATC

MMP-2 R: GCTGGTTAACTACAGAGGAGGACAG (146 bp);

MMP-9 F: GACGACGACGAGTTGTGGTCGCT,

MMP-9 R: CGTCTGTGGTGCAGGCCGAATAG (130 bp);

MT1 F: AGAGCCAGGGTATCCCAAGT,

MT1 R: AACGCTGGCAGTAAAGCAGT (250 bp);

GAPDH F: TTGTCAAGCTCATTTCCTGGTATG,

GAPDH R: GGATAGGGCCTCTCTTGCTCA (143 bp);

β- Actin F: GGGACCTGACAGACTACCTCATG,

β-actin R: GAGGATGCGGCAGTGGC (161 bp).

### Immunocytochemistry and Cytoskeleton Labelling

Cortical neurons or astrocytes were rinsed in phosphate buffer saline (PBS) and fixed in 4% paraformaldehyde (PFA) for 10 min. For immunocytochemistry, cells were pre-incubated with 0.1% triton X-100, 3% bovine serum albumin (BSA, Sigma-Aldrich) and 5% normal goat serum (NGS) (Jackson ImmunoResearch, West Grove, PA, USA) for 60 min, followed by 90 min incubation at room temperature with the following primary antibodies: mouse anti-βIII-tubulin, (1/500, Sigma-Aldrich), rabbit anti-Glial Fibrillary Acidic Protein (GFAP) (1/400, Dako, Glostrup, Denmark), mouse anti-phosphorylated Tau-1 (1/200, Sigma-Aldrich), chicken anti-MAP2 (1/10000, Abcam, Cambridge, UK) goat anti-MMP-9 (1/100, R&D Systems) or goat anti-MMP-9 (1/100, Santa Cruz Biotechnology, Santa Cruz, CA, USA), rabbit anti-MMP-2 (1/200, Chemicon, Temecula, CA, USA), mouse anti-Green Fluorescent Protein (GFP) (1/500, Roche Diagnostics, Mannheim, Germany), and rabbit anti-ser3 p-cofilin (1/100, Santa Cruz Biotecnology). Goat anti-TIMP-1 (1/100, R&D Systems) was incubated overnight at 4°C. The antibodies were diluted in PBS containing 0.1% triton X-100, 3% BSA, and 5% NGS. The cells were then rinsed in PBS, incubated for 1 h with Alexa Fluor® 488–594, antibodies (1/600–800, Molecular Probes, Eugene, OR, USA) or with biotinylated anti-goat antibody (1/400, Jackson Immunoresearch) followed by 1 h additional incubation with Alexa Fluor® 488 streptavidin (1/400, Molecular Probes) to reveal MMP-9 immunostaining, and 0.5 µg/ml of nuclear marker Hoechst #33258 (Molecular Probes). For double labelling experiments, the anti-βIII-tubulin and anti-GFAP antibodies or the anti-Tau-1 and anti-MAP2 antibodies were incubated together and the secondary antibodies used as indicated above. For F-actin and nuclear labelling, cells were incubated after immunocytochemistry with Texas Red-X phalloidin (Molecular Probes), 0.5 µg/ml Hoechst #33258 in 0.1% triton, 3% BSA and 5% NGS for 1 h at room temperature, rinsed in PBS, and mounted in fluorescence mounting medium (Dako). Cells were observed under a Nikon E800 upright microscope equipped with epifluorescence and TRITC, FITC and DAPI filters, and images were analysed using an Orca-ER CCD camera (Hamamatsu Photonics, Massy, France) and the LUCIA image analysis software (Laboratory Imaging, Prague, Czech Republic).

### Morphological Analyses

In each experiment, fluorescence microphotographs of at least 5 randomly selected fields per well and 3 wells per experimental condition were taken, and the following parameters were analysed: the total number of cells was determined on the basis of nuclear Hoechst staining and ratios of βIII-tubulin or GFAP positive cells determined from combined immunocytochemistry of neuronal or glial markers. The neuronal processes and growth cones of all βIII-tubulin positive cells were manually drawn using a computer mouse. The mean length of the neuritic arbour was obtained by dividing the total length of neurites in a field by the number of cells immunoreactive for βIII-tubulin. At 48 h post-seeding, the same procedure was used to determine the length of dendrites and axons by measuring MAP2 and Tau-1 immunoreactive neurites, respectively. The number of neurites per neuron was used as an indication of branching and network complexity. A neurite segment was defined as the distance between branching points or the distance between the branching point and the tip of the neurite. The mean number of neurite segments per neuron was calculated from the number of segments per microscopic field divided by the number of βIII-tubulin positive cells. The relative frequency of neurites of a given length was classified by intervals of 10 µm. The number of growth cones per βIII-tubulin positive cell, their shape, area and perimeter were analysed after fluorescence labelling with Texas Red-X phalloidin. To test whether the shape of growth cones was related to their dynamics, the index of circularity or “f circle” (4 pi x area/perimeter crofton2) was calculated. This parameter describes the degree to which a shape differs from a circle and it varies from 0 to 1 (a perfect circle  = 1). We analysed around 900 neurons per experiment.

### Gel Zymography

Gel zymography was performed as previously described [6]. Culture supernatants were collected and protein concentrations normalised using the Lowry method (Bio-Rad, Hercules, CA, USA). Equal amounts of protein were subjected to 8.5% SDS-PAGE (Bio-Rad) containing porcine gelatin at 4.5 mg/ml (Sigma-Aldrich) in non-denaturing, non-reducing conditions and using a MiniBlot system (Bio-Rad). Gels were washed twice for 30 min in 2.5% Triton X-100 to remove SDS, and incubated for 48 h in 50 mM Tris pH 7.5, 10 mM CaCl_2_, at 37°C. Gels were then stained with 0.1% Coomassie blue G-250 (Bio-Rad) for 3 h in 30% ethanol and destained with a solution containing 5% acetic acid until clear bands of gelatinolysis appeared on a dark background. Gels were digitised and optical densities assessed with the Bio 1D software (Vilber Lourmat, Marne-la-Vallée, France). Some zymogram gels were incubated with 1 mM 1,10-*0*-phenanthrolin, a broad spectrum inhibitor of metalloproteinases. Human recombinant active MMP-2 (hrMMP-2) (100–200 pg, Chemicon) was used as a positive and normalising control.

### Reverse Gel Zymography

Reverse gel zymography was performed according to the method previously reported [29], [30]. Culture supernatants and lysates were collected and protein concentrations were normalised as mentioned above. Equal amounts of protein were subjected to 8.5% SDS-PAGE containing 2.25 mg/ml porcine gelatin and 160 ng/ml of human recombinant active MMP-9 (Chemicon). Samples were loaded in Laemmli buffer and run at constant amperage (40 mA). The gels were incubated on a rotary shaker for 3 h in 2.5% Triton X-100. The Triton X-100 solution was replaced with MMP activating buffer (50 mM Tris-HCl, pH 7.5, 10 mM CaCl_2_) at 37°C for 48 h. Gels were then processed for analysis as described above.

### Isolation of Intracellular, Cytoskeletal and Membrane Fractions

Subcellular fractioning was performed on cortical neurons using the ProteoExtract subcellular proteome extraction kit (Calbiochem) according to the manufacturer's instructions. Proteins were extracted with different buffers from major subcellular compartments and on the basis of the difference in protein solubility. The first cytosolic fraction was obtained by incubating the cells with an extraction manufacturer's buffer at 4°C for 10 min. A second fraction contained the cell membrane and organelles, and centrifugation of this fraction allowed separation of membrane (supernatant) and cytoskeletal (pellet) fractions. Equal volumes of each fraction samples were resolved by SDS-PAGE, and gel zymography was performed as described above.

### Extraction of Gelatinases and Western Blot

Culture supernatants from cortical neurons were collected 24 h after seeding. For gelatinase extraction, supernatants were concentrated using centrifugal filter devices (Amicon Ultra, Millipore, Billerica, MA, USA) at 7500 x g during 15 min at 4°C, and were incubated in the presence of 60 µl of gelatin-sepharose 4B (Amersham Biosciences, Buckinghamshire, UK) overnight. Gelatin-sepharose 4B was eluted with working buffer (50 mM Tris-HCl, pH 7.6, 150 mM NaCl, 5 mM CaCl_2_, 0.005% Brij-35) and 10% DMSO at room temperature. Protein concentrations were determined using the Lowry method. Equal amounts of protein in Laemmli buffer containing 10% β-mercaptoethanol were boiled and separated by 8% SDS-PAGE. mrTIMP-1 was loaded as positive control. Proteins were transferred onto nitrocellulose membranes (Amersham Biosciences) in transfer buffer (25 mM Tris, 192 mM glycine, 20% ethanol). Membranes were incubated overnight in blocking buffer at room temperature and then probed with rabbit anti-MMP-2 (1/500, Chemicon); goat anti-TIMP-1 (1/100, R&D Systems); mouse anti-GFP (1/500, Roche Diagnostics); mouse anti-MAP2 (1/500), mouse anti-βIII-tubulin (1/500) and mouse anti-β-actin (1/3000), all three from Sigma-Aldrich; and rabbit anti-ser3 p-cofilin (1/500, Santa Cruz Biotecnology). All antibodies were diluted in blocking buffer (Roche Diagnostics). After incubation with primary antibodies, membranes were incubated with a peroxidase conjugated secondary antibody (Jackson Immunoresearch). Finally, proteins were detected using a chemiluminescence kit (Roche Diagnostics) and films were digitised and analysed using the Bio 1D software.

### TIMP-1-GFP Construct and Transfections

Full length mouse TIMP-1 coding regions were amplified by PCR from mouse brain cDNA produced as described above, using the following primers containing unique restriction sites in their 3′ end:

TIMP-1/GFP-F: ATATATGAATTCTAAATGATGGCCCCCTTTGCATCTCTG (EcoR1),

TIMP-1/GFP-R: ATATATGGATCCCGGGCCCCAAGGGATCTCCAAGT (BamH1).

The TIMP-1 PCR product was cloned in frame with GFP in the EGFP (N1) vector (Clontech, Mountain View, CA, USA) digested with the same enzymes beforehand and the plasmid construct was fully sequenced on both strands.

Primary cortical neurons were seeded as indicated above. After 18–20 h in culture, the plating medium was aspirated and cells were transfected using 1 µg of plasmid DNA encoding GFP or the TIMP-1/GFP construct mixed with 5 µl of lipofectamine (Invitrogen) and 1 µl of combimag beads (Magnetofection Kit OZ Biosciences, Marseilles, France), according to the manufacturer's protocol. The mix was incubated for 30 min on a magnetic plate (OZ Bioscience) at 37°C and then replaced by pre-warmed serum-free defined medium for 48 h until fixing in 4% PFA. N2a cells were transfected using jetPEI (Qbiogene, Carlsbad CA, USA) in Opti-MEM medium as recommended by the manufacturer. Briefly, 1 µg of plasmid DNA encoding GFP or the TIMP-1/GFP construct was mixed with 100 µl of NaCl 150 mM, 2 µl of jetPEI and applied to 50000 cells plated on 12 mm diameter glass coverslips 24 h after seeding. The supernatants of these cells were collected 48 h after and used to assess the extent of TIMP-1/GFP inhibition on MMPs. The transfection efficacy was >30%.

### Fluorigenic Assay on mrTIMP-1 Inhibitory Activity

The inhibitory activity of mrTIMP-1 on active hrMMP-2 was evaluated using a fluorimeter (DTX 800 Multimode Detectors, Beckman Coulter, Fullerton, CA, USA). The cleavage of gelatinase-specific quenched fluorescence substrate Mcmat (0.5 µg) (Calbiochem), was measured as arbitrary fluorescence units, using 325 nm excitation and 400 nm emission filters at 37°C for 1 h. Twenty ng of active hrMMP-2 was used and the inhibitory activity of 100 ng of mrTIMP-1 tested for 1 h. The control contained 50 mM Tris/HCl, 10 mM CaCl_2_, 100 mM NaCl, pH 6,8 (TCN buffer) and substrate, but no proteinase. A second control was used to discard a possible inhibitory effect of the TNC buffer in which mrTIMP-1 was diluted.

### Statistical Analysis

Data were analysed using the analysis of the variance (ANOVA), followed by either post-hoc Student-*t*, Tukey's or Dunnett's test. Statistical significance was achieved at p≤0.05.

## Results

### TIMP-1 Content in Cortical Neurons

**Figure 1:**
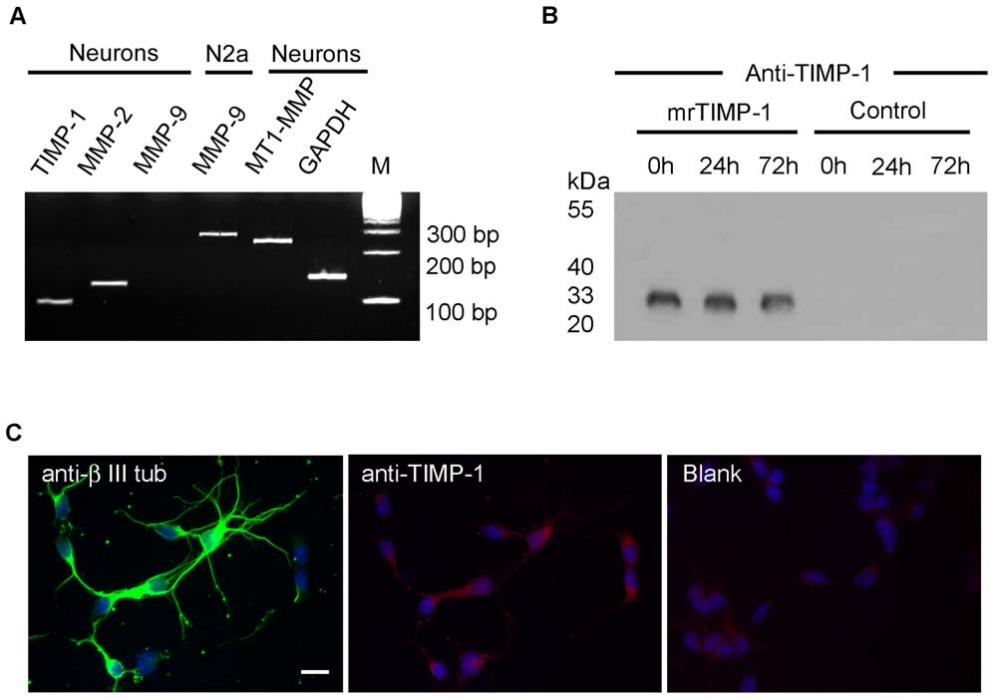
Expression of TIMP-1 and MMPs in primary cortical neurons. **A.** Semi-quantitative RT-PCR followed by separation of the PCR products in ethidium bromide stained gels showing expression by cortical neurons 24 h after seeding and by N2a neuroblastoma cells of mRNAs encoding endogenous TIMP-1, MMP-2, MMP-9 and MT1-MMP after 35 cycles of PCR. Note that in cortical neurons, MMP-9 mRNA is barely detected as compared with N2a cells and with the other neuronal mRNAs which were already detected at 30 cycles of PCR. GAPDH (30 cycles) was used as a standard; 100 bp molecular weight ladder (M). **B.** Western blot showing TIMP-1 immunoreactivity after equal protein loading from supernatants of cortical neurons at different times after adding 2.5 nM of mrTIMP-1, or from control untreated cultures at the same time points, 0, 24 and 72 h. Note that endogenous TIMP-1 is not detected in untreated cultures, whereas mrTIMP-1 is easily detected in the supernatants of these cultures even after 72 h, indicating high stability of the protein. **C.** Fluorescent microphotographs of cortical neurons showing βIII-tubulin (green) and TIMP-1 (red) immunostaining co-labelled with the nuclear marker Hoechst (blue) 24 h after seeding. Note low levels of endogenous TIMP-1, slightly above background levels (Blank) only in the soma of some cells. Scale bar 20 µm. Figures are representative of at least 3 independent experiments.

Semi-quantitative RT-PCR was performed on mRNAs encoding TIMP-1, MMP-2, MMP-9, MT1-MMP, GAPDH (Fig. 1A), β-actin (not shown) and 18S RNA (not shown), the latter 3 being used to standardise the experiments. PCR products were analysed after 30 (not shown), 33 (not shown) and 35 cycles (Fig. 1A). While TIMP-1, MMP-2, MT1-MMP and GAPDH PCR products were already detected at 30 cycles, the MMP-9 PCR product was barely detected even after 35 PCR cycles in cortical neurons cultured *in vitro* for 24 h. At 39 cycles the MMP-9 PCR products was clearly visible, but additional non specific products of weak intensity appeared as well (not shown). The efficiency of the selected PCR primers for MMP-9 was established by amplification of the appropriate PCR product in N2a neuroblastoma cells (Fig. 1A) and astrocytes (not shown). We thus conclude that in cortical neurons cultured for 24 h MMP-9 mRNA is expressed at significantly lower levels than the mRNAs encoding the other proteins of interest in this study. Although TIMP-1 mRNA was detected using RT-PCR approaches (Fig. 1A), the protein was undetectable by western blot in the supernatants of our cultures 24 h after seeding (Fig. 1B) and immunolabelling was just slightly above background levels in a few neurons (Fig. 1C). These data are in agreement with previous reports showing low levels of TIMP-1 in physiological conditions [4], [5]. In contrast with the low amounts of TIMP-1 found in cortical neurons, the supernatant of control or LPS-treated cortical astrocytes accumulated in 24 h between 10 and 100 ng/ml (∼0.3–3 nM) of TIMP-1, as demonstrated by western blot (not shown). Astrocytes also displayed intense immunolabelling for TIMP-1 (not shown). Similar concentrations of TIMP-1 have been reported in the cerebrospinal fluid of patients suffering from various neurodegenerative disorders [31]. Together, these data determined the mrTIMP-1 working concentrations in the present study and suggested that the low levels of endogenous TIMP-1 would not interfere with exogenously added mrTIMP-1. The latter was still easily detected by western blot in the supernatants 0, 24 and 72 h after exposure, indicating a good stability of the recombinant protein in culture media conditioned by neurons (Fig. 1B). The MMP inhibitory effect of mrTIMP-1 was confirmed by the inhibition of MMP-2 dependent cleavage of a fluorigenic substrate, as assessed by spectrofluorescence (Fig. S1). Overall, the present experimental model tentatively recreates the inflammatory/post-lesion situation where most TIMP-1 is released into the neuronal microenvironment by reactive astrocytes that express high levels of TIMP-1 compared to control, as previously reported [4], [7].

### Effects of mrTIMP-1 on Cell Culture Composition and Viability

**Figure 2:**
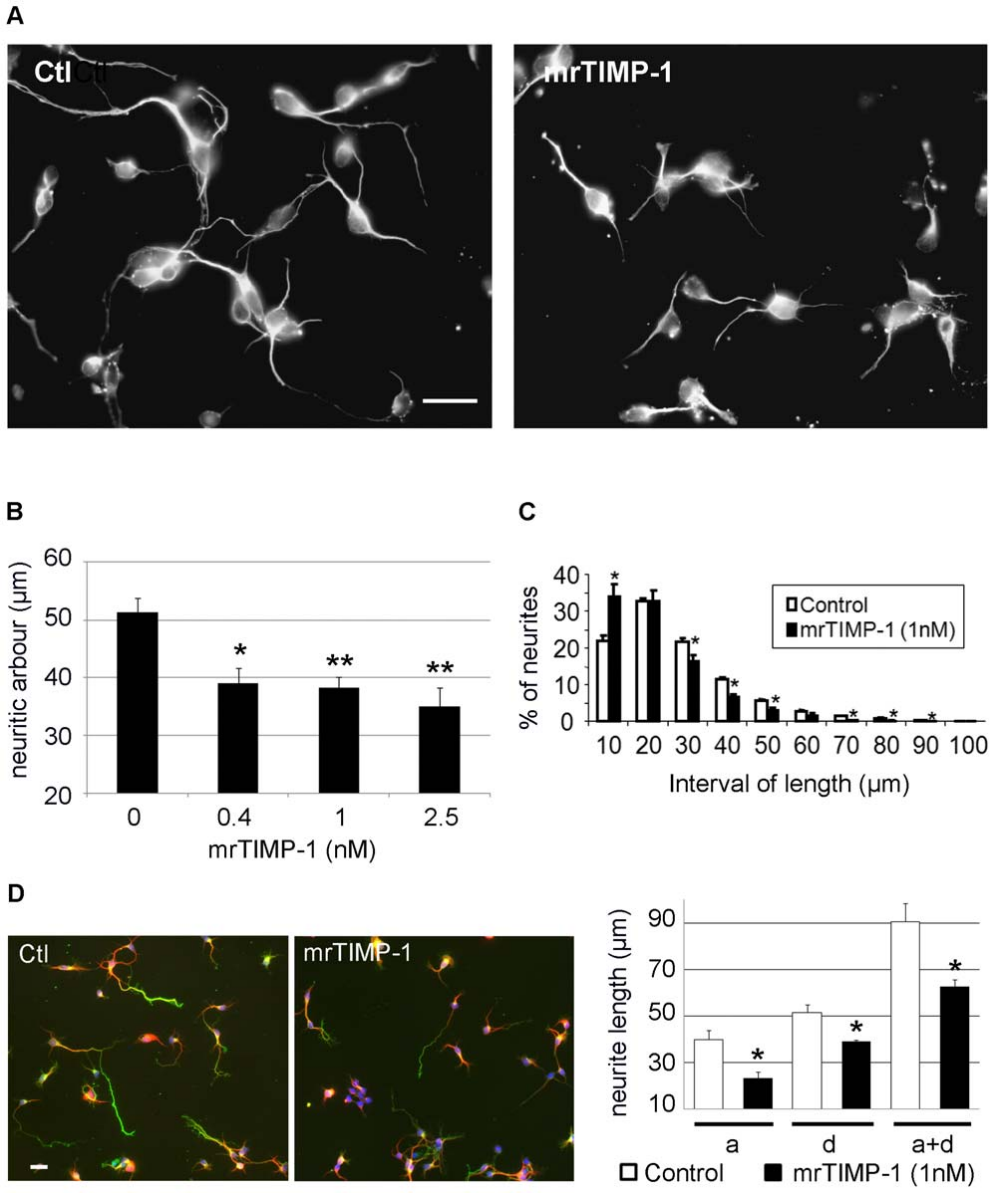
Effects of mrTIMP-1 on neurite outgrowth of cortical neurons 24 and 48 h after seeding. **A.** Fluorescent microphotographs of cortical neurons showing βIII-tubulin staining 24 h after seeding. Note that the length of neurites decreases after exposure to mrTIMP-1 (1 nM in the example). **B.** Quantification of these reductions after 24 h exposure to several concentrations of mrTIMP-1. **C.** Quantification by intervals of length, indicating that mrTIMP-1 induces an important increase in the proportion of small neurites (between 0 and 10 µm), whereas longer neurites are significantly more frequent in the control cultures. **D.** Fluorescent microphotographs of cortical neurons showing immunostaining for Tau-1 (green) and MAP2 (red), and Hoechst labelling (blue) 48 h after seeding. The graph represents the length of axons (a), dendrites (d) and the total neuritic arbour (a+d). Note that 48 h of treatment with mrTIMP-1 significantly reduced the size of both axons and dendrites. Values represent the means ± SEM of 3–8 independent experiments. *p<0.05, **p<0.01, ANOVA followed by Dunnett's (graph B) and Student-*t* (graphs C and D) tests. Scale bars 20 µm.

Twenty four hours after seeding, untreated cultures were composed of 88.6% of βIII-tubulin positive cells with processes (considered as differentiated neurons), 6.4% of βIII-tubulin positive cells without neurite extensions (non-differentiated neurons), 2.97% of GFAP positive cells and 2.03% of unidentified cells negative for both neuronal and astrocytic markers (see Table 1). Moreover, only a few neurons exhibited an incipient axonal differentiation as assessed by double immunostaining with anti-Tau-1 and anti-MAP2 antibodies, an axonal and a dendritic specific marker, respectively. Virtually all neurons exhibited a colocalised distribution of Tau-1 and MAP2 (Fig. S2) and only 11% of them displayed a slightly higher intensity of labelling for Tau-1 in one of their neurites, which coexisted with conspicuous MAP2 labelling. This clearly indicated a poor degree of axo-dendritic differentiation at this stage in serum free pure neuronal cultures, unlike cultures based on methods such as Banker's, where co-cultured astrocytes provide neurons with the necessary trophic support for early axo-dendritic differentiation [32], [33]. In contrast with 24 h cultures, many neurons displayed a discrete distribution of MAP2 and Tau-1 immunostaining at 48 and 72 h post-seeding (Fig. 2D and Fig. S2, respectively), suggesting axo-dendritic differentiation. Therefore, unbiased statistical evaluation of mrTIMP-1 effects on the length of dendrites and axons was possible at 48 h when the density of axo-dendritic circuitry allowed for individual tracing of neurite extensions (Fig. 2D).

**Table 1:** Effects of mrTIMP-1 on the content of cortical cultures 24 h after seeding.

mrTIMP-1 (nM)	0	0.4	1	2.5
**# Hoechst positive cells**	60±4.8	63±6.7	64±5.8	62±3.3
**% astrocytes**	2.9±0.2	n.a.	6.2±0.4*	3.5±0.4
**% differentiated neurons**	89±0.7	85±2.2	86±1.2	87±1.1
**% non differentiated neurons**	6.4±0.7	8.5±2.2	7.1±1.2	6.7±0,9

mrTIMP-1 induced no changes in the number of viable cells or in the number of differentiated or non differentiated neurons. However, it augmented the number of GFAP positive cells. Values represent the means ± SEM of 4–8 independent experiments * p<0.01, ANOVA followed by Dunnett's test.

The number of cells in control cultures, counted as Hoechst positive nuclei, remained constant across 10 independent experiments (59.7±4.8 cells per microscopic field of 87 mm^2^). This number of viable cells was not affected by 24 h exposure to mrTIMP-1 at any concentration studied and the treatment did not induce any sign of cytotoxicity such as chromatin compaction or neurite breakage, and did not alter the ratio of differentiated and non-differentiated βIII-tubulin positive cells (Table 1).

Twenty four hours following exposure to 1 nM mrTIMP-1, the number of astrocytes doubled (Table 1) as compared with untreated control cultures. Although these experiments were not set up to study the effects of TIMP-1 on astrocytes, which represent a very low percentage of cells, the present results are in keeping with previous observations in primary cultures of cortical astrocytes, where TIMP-1 increased [^3^H] thymidine uptake by 200% [13].

### Effects of mrTIMP-1 on Neurite Length in Cortical Neurons

Previous work in our laboratory suggested the implication of TIMP-1 in the control of axonal remodelling *in vivo* [16] and prompted us to investigate more specifically the effects of mrTIMP-1 on cultured neurons. Twenty four hours of exposure to mrTIMP-1 was considered as a minimum time for TIMP-1 to act in neurons after pathological driven upregulation. The length of neurites, measured in βIII-tubulin labelled cells, was significantly reduced between 25 and 30% after mrTIMP-1 exposure (Fig. 2A and B). In addition, as illustrated in figure 2C for the 1 nM concentration, the majority of the smallest neurites [0; 10 µm] were observed in cultures treated with mrTIMP-1. This proportion was similar between control and mrTIMP-1-treated cultures in the next length interval [10; 20 µm] and was inverted in the [20; 30 µm] interval and thereafter, with control cultures displaying a higher number of the longer neurites. In addition, the longest neurites were systematically found in control cultures. The effects of mrTIMP-1 on the distribution of length frequencies were similar regardless of the concentration studied: 0.4 nM (n = 4), 1 nM (n = 4), 2.5 nM (n = 8). The inhibitory effects of mrTIMP-1 on neurite outgrowth were still present after 48 h treatment and affected equally both dendrites and axons (Fig. 2D)

### Effects of mrTIMP-1 on Actin Cytoskeleton and Growth Cone Morphology

**Figure 3:**
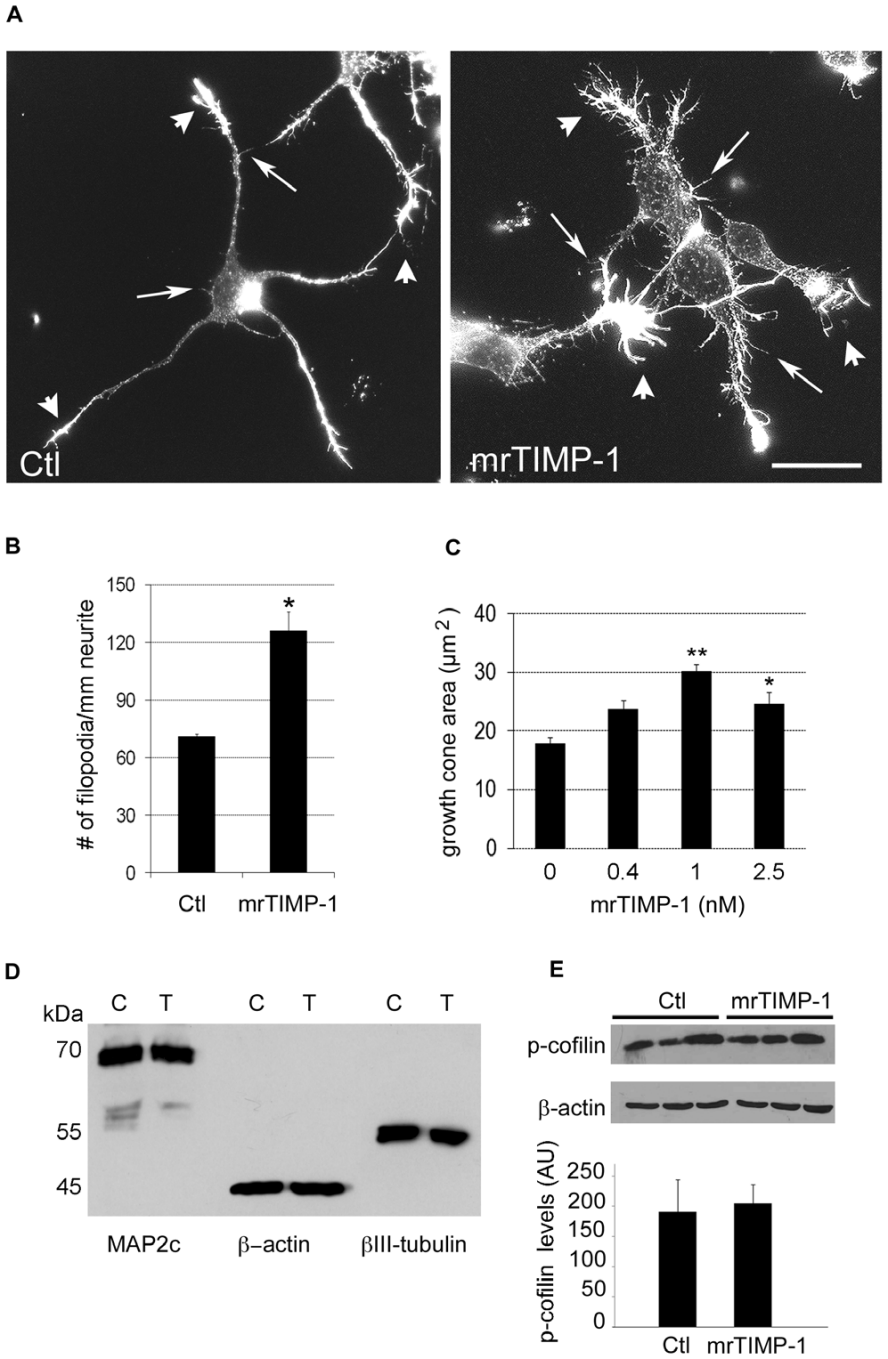
Effects of mrTIMP-1 on the morphology of cortical neurons and expression of cytoskeletal proteins. **A.** Fluorescent microphotographs of cultured cortical neurons labelled with Texas Red-X phalloidin, showing increases in the size of growth cones (arrowheads) and the number of F-actin rich filopodia (arrows) after 24 h of mrTIMP-1 treatment (1 nM) as compared with control cultures. **B** and **C.** Quantification of these changes showing significant increases in the number of filopodia/mm of neurite (B), and the area of growth cones (C) after mrTIMP-1 treatment. **D.** Western blot from lysates of cortical neurons treated with 1 nM mrTIMP-1 for 24 h showing no differences between control (C) and mrTIMP-1 treated (T) cultures for different cytoskeleton proteins (MAP2c, β-actin and βIII-tubulin). **E.** Western blot and quantification of phospho-cofilin levels from lysates of cortical neurons (3 independent experiments). No changes were observed after treatment with 1 nM mrTIMP-1 for 24 h. β-actin was used as a control of equal protein loading. Images in A, B and D are representative of results obtained in 4 independent experiments. Values in B and C represent the means ± SEM of 4 independent experiments. *p<0.05, ** p<0.01, Student-*t*-test (B); Dunnett's test (C). Scale bar 20 µm.

To assess changes in the morphology of growth cones and other actin rich structures, we stained F-actin using Texas Red-X phalloidin. Microscopic observation revealed that cultures exposed to mrTIMP-1 for 24 h exhibited a less organised actin network and an increased number of actin rich filopodia along the neurite shaft (Fig. 3A). Indeed, the number of these protrusions nearly doubled as compared to control cultures (Fig. 3B). Moreover, the area of leading growth cones augmented almost 2-fold (Fig. 3C) after exposure to mrTIMP-1. No significant changes were observed in the index of circularity (not shown).

### mrTIMP-1 Did Not Alter the Levels of Cytoskeletal Proteins

We next sought to examine whether morphological changes would be supported by changes in the levels of cytoskeletal proteins such as β-actin, β-III-tubulin, or MAP2c, a regulator of microtubule assembly. Using western blot approaches, no changes were observed in their expression levels after 24 h treatment, suggesting that mrTIMP-1 effects on cortical neuron morphology rather result from redistribution of cytoskeletal components (Fig. 3D). In order to further ascertain a possible link between TIMP-1 effects and a signalling cascade in relation with actin polymerisation and depolymerisation, we sought for changes in the phosphorylation of cofilin, an actin binding protein that depolymerises actin and looses this capacity upon phosphorylation. Using an anti-ser 3 p-cofilin antibody, previously reported to allow for detection of subtle changes in cofilin phosphorylation [34], we could not detect significant modifications in the levels of phosphocofilin after mrTIMP-1 treatment either by western blot (Fig. 3E) or by immunofluorescence (results not shown).

### Effects of Wild Type and Mutated Inactive Forms of Truncated N-Terminal TIMP-1 on Cortical Neurons

**Figure 4:**
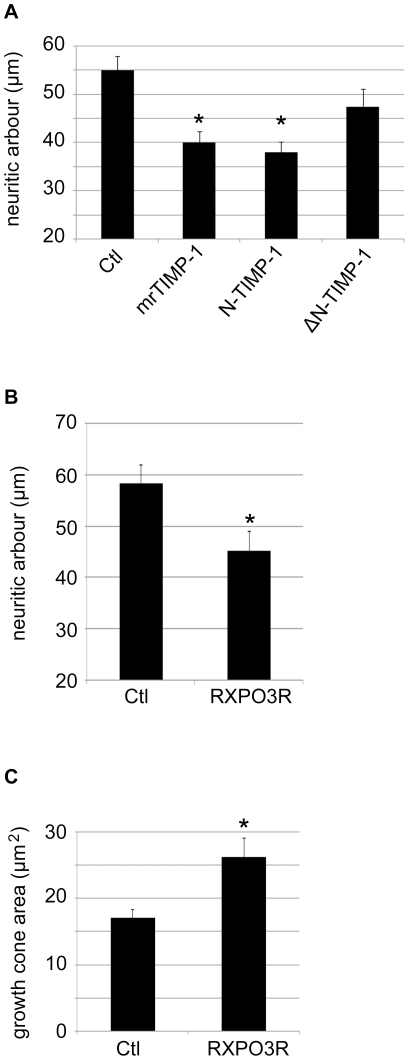
Inhibition of neurite outgrowth by the N-terminal domain of TIMP-1. **A.** Plot representing the average length of neurites per neuron after 24 h treatment with 2.5 nM of mrTIMP-1, the wild type form of the N-terminal domain of TIMP-1 (N-TIMP-1) or the mutated inactive form of the latter (ΔN-TIMP-1). **B** and **C.** Plots representing the effects of RXPO3R (2 µM) on the length of neurites (B) and the area of the growth cone (C). The values represent the means ± SEM of at least 3 independent experiments. *p<0.05, ANOVA followed by Tukey's (A) or Student-*t* (B and C) test.

Both the N-terminal and C-terminal domains of TIMP-1 influence its biological functions. The N-terminal domain interacts with the active site of the target MMP and carries the MMP inhibitory activity, whereas the C-terminal domain conveys the binding to pro-MMP-9 and protein-protein interactions that confer to TIMP-1 functions independent of MMP inhibition [2]. In order to distinguish between these two possibilities, we investigated the effects on cortical neuronal cultures of wild type (NT1) and mutated (ΔNT1) truncated N-terminal forms of TIMP-1 after 24 h exposure. The NT1 form of the protein preserves the MMP inhibitory activity of full length TIMP-1 against MMP-1, MMP-2, MMP-3 and MMP-9 and a relatively poor inhibitory activity against MT1-MMP. These inhibitory capacities are completely abrogated in the ΔNT1 mutated form with the exception of MMP-9 inhibition which is selectively preserved [35], [26]. In the presence of 2.5 nM NT1, neurite length was significantly reduced by 32%, whereas the ΔNT1 mutated form did not induce significant changes with respect to the control group (Fig. 4A). These observations clearly indicate that TIMP-1 effects on the morphology of cortical neurons are predominantly mediated by the inhibition of MMP activity.

### Effects of Broad Spectrum Specific MMP Inhibitor

In order to confirm that the MMP inhibitory properties of the N-terminal domain of TIMP-1 modulate neuronal morphology, we next asked whether broad spectrum MMP inhibitors would mimic TIMP-1 effects. We used RXPO3R (2 µM), a pseudophosphinic MMP inhibitor which does not inhibit the closely related ADAM family of metalloproteinases [23], [16]. Like the full length mrTIMP-1 and its NT1 variant, 24 h of exposure to RXPO3R significantly reduced the length of neurites and increased the area of growth cones (Fig. 4B and C), without affecting cell viability or the proportion of differentiated neurons (not shown). The convergence of the effects induced by TIMP-1, NT1 and RXPO3R on the morphology of cortical neurons further supports the idea that TIMP-1 effects are mainly mediated by its inhibitory activity on MMPs.

### Effects of a TIMP-1/GFP Fusion Protein Expressed in Cortical Neurons

**Figure 5:**
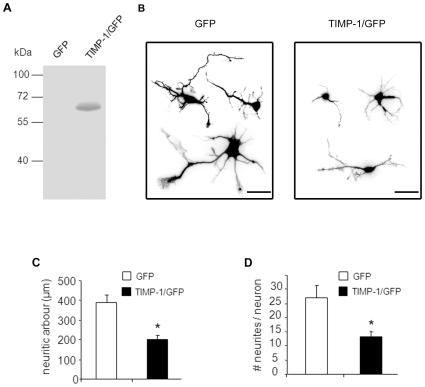
Effects of TIMP-1/GFP fusion protein on the morphology and neurite development of cortical neurons. **A.** Reverse gel zymography from supernatants of N2a cells transfected with TIMP-1/GFP fusion protein demonstrating that TIMP-1/GFP retains inhibitory activity on MMP proteolytic activity. Note that the band of inhibited MMP-9 activity corresponds to the size of TIMP-1 (33 kDa) + GFP (28 kDa). **B.** Examples of cortical neurons transfected with GFP or TIMP-1/GFP and immunostained with an anti-GFP antibody 48 h after transfection. White fluorescence photomicrographs were converted to black negative images to improve the details on the neuritic arbour. Note that TIMP-1 overexpression in these neurons strongly reduces the complexity of their arborisation. Scale bar 20 µm. **C and D.** Quantification of the changes induced by TIMP-1/GFP overexpression compared to GFP alone. The values represent the means ± SEM of 4 independent experiments. *p<0.05, Student-*t*-test.

The data above demonstrate that exogenous mrTIMP-1 affects neuron morphology and hence recreate a paracrine action of TIMP-1 when released, for instance, by reactive surrounding glia in different pathological settings. However, inflammation driven expression of TIMP-1 by glia is normally preceded by the induction of TIMP-1 expression in cortical and hippocampal neurons resistant to degeneration [4], [6]. Therefore, we next asked whether TIMP-1 also elicits autocrine effects on neuron outgrowth. To address this question, we generated a plasmid construct encoding a mouse TIMP-1/GFP fusion protein that retains the inhibitory activity of TIMP-1 on MMPs, as demonstrated by reverse zymography on supernatants from N2a transfected cells (Fig. 5A). Forty eight hours after transfection the transgene was homogeneously distributed in all cell compartments of both βIII-tubulin positive (Fig. 5B) and negative cells (not shown). The first represented 2.02% of total cells in the culture, whereas βIII-tubulin negative cells (presumably astrocytes) accounted for 0.5%. These percentages reached 2.06% and 16.6% when the number of TIMP-1/GFP transfected cells was respectively referred to the number of neuronal or non-neuronal cells, clearly indicating a higher transfection rate for the latter. Western blot (Fig. S3) using anti-GFP antibody did not reveal a detectable presence of TIMP-1/GFP in the supernatants of transfected cultures. In these conditions, as illustrated in figures 5C and 5D, TIMP-1/GFP overexpression strongly reduced neurite length (48.3%) and the number of neurites (51.8%). We also attempted to evaluate the effects of TIMP-1/GFP overexpression on actin cytoskeleton, but after nearly 70 h in culture transfected cells were not longer individualised and their extensions and growth cones were intermingled with surrounding neurons (Fig. S4). The few transfected and isolated neurons found in each experiment (only 3–4 per well for each experimental group) were not sufficient to reveal significant changes on the size of growth cones after TIMP-1/GFP overexpression.

### Expression of Potential TIMP-1 Targets, MMP-2 and MMP-9, and Effects of MMP-2 Selective Inhibition

**Figure 6:**
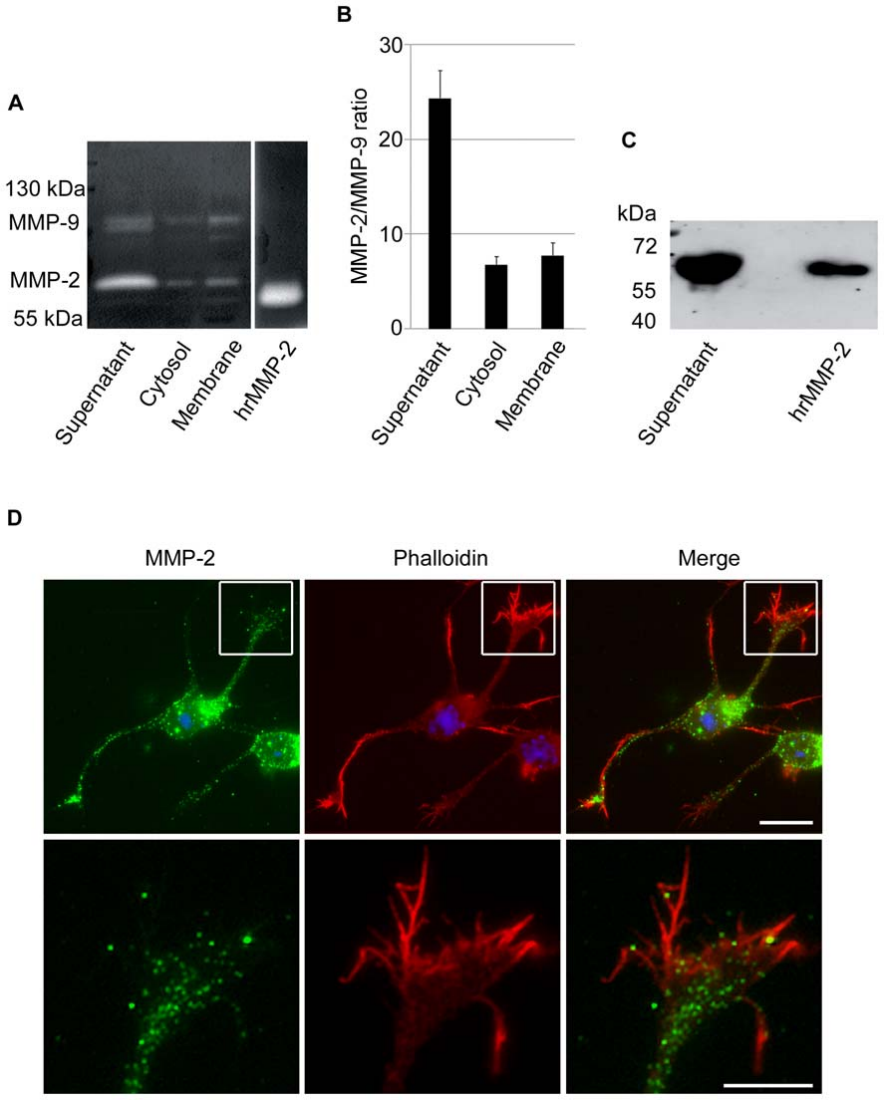
Expression of MMP-2 and MMP-9 in cortical neurons 24 h after seeding. **A.** Gelatin zymograms of MMP-2 and MMP-9 from supernatant of cortical neurons, cytosol and membrane fractions. Human active recombinant MMP-2 (hrMMP-2) was used as a positive control (200 pg). The zymogram shows much higher expression of MMP-2 (∼70 kDa) than MMP-9 (∼100 kDa) in the supernatant, higher expression of MMP-2 in the cytosol where MMP-9 is barely detectable, and rather similar intensity of gelatinolytic bands for both gelatinases in the membrane fraction. **B.** Quantification of the MMP-2/MMP-9 ratio from zymograms, where active recombinant MMP-2 was used as normalising control. Values represent the means ± SEM of 3 independent experiments. Note that the quantification takes into consideration a 5-fold higher gelatinolytic activity of MMP-9 than MMP-2. **C.** Western blot demonstrating MMP-2 immunoreactivity in the supernatants of cortical neurons after enrichment and precipitation of the samples with gelatin beads. Active human recombinant MMP-2 (5 ng) was used as a positive control. Images are representative of 3 independent experiments. **D.** Fluorescence microphotographs showing immunolabelling of MMP-2 (green) and phalloidin F-actin labelling (red) in cortical neurons 24 h after seeding. Hoechst #33258 stained the nuclei (blue). Note that MMP-2 is distributed in the cell body and neurites. In the growth cones, MMP-2 is mainly located to the central domain (insets with high power magnifications in the lower row) and virtually excluded from F-actin rich areas, notably the peripheral domain. Scale bars are 20 µm for entire neurons and 10 µm for close up of growth cones.

**Figure 7:**
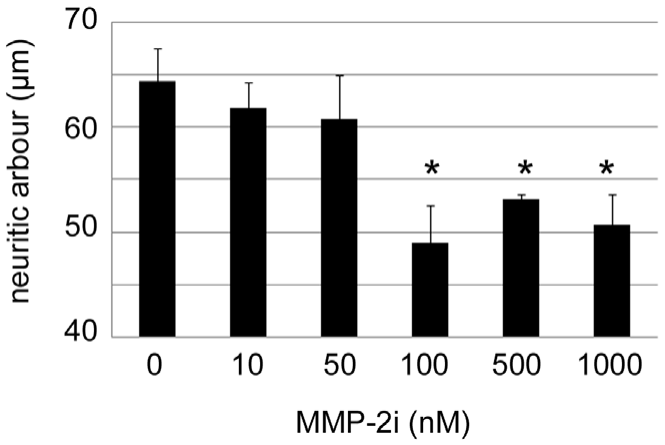
Selective MMP-2 inhibition reduces neurite length. Plot representing the average length of neurites per neuron after 24 h treatment with a selective MMP-2 inhibitor. The reduction of neurite length is dose-dependent. The values represent the means ± SEM of 4 independent experiments. *p<0.05, Dunnett's test.

Since RXPO3R and TIMP-1 are very poor inhibitors of ADAMs and MT-MMPs, respectively, it is plausible that their effects on neuron growth and morphology result from the inhibition of secreted MMPs synthesised by cortical neurons. Gelatinases MMP-2 and MMP-9 are potential targets for TIMP-1. The MMP-2 mRNA was clearly more abundant than MMP-9 mRNA and this ratio was preserved at the protein level. Indeed, MMP-2 gelatinolytic activity (∼70 kDa) was consistently displayed in the supernatant, cytosolic and membrane fractions of cultured neurons. MMP-9 gelatinolytic activity (∼100 kDa) was relatively faint in the supernatant and occasionally detected in the cytosol. Both proteinases were detected in the membrane fraction, which displayed a more conspicuous band of lower molecular weight, as compared with the supernatant and cytosolic fractions (Fig. 6A). Considering that MMP-9 has a 5-fold higher gelatinolytic capacity than MMP-2, we found that the latter was between 6-fold and 24-fold more abundant than MMP-9 in the tested fractions (Fig. 6B). The expression of MMP-2 by cortical neurons was further confirmed by western blot (Fig. 6C) and immunocytochemistry (Fig. 6D). The levels of MMP-9 were not sufficient to be unequivocally detected with two different antibodies by western blot or immunocytochemistry in any of the experiments we performed (not shown). In contrast, MMP-2 immunolabelling appeared to be punctate in the cell body and neurites and often conspicuous in the growth cone (Fig. 6D). MMP-2 immunoreactivity was mainly observed in the central domain of the growth cone and generally excluded from the more motile peripheral domain rich in F-actin (Fig. 6D). Provided that MMP-2 was constitutively expressed by cortical neurons and in particular in areas that are essential for neurite outgrowth, we tested whether the latter was affected by selective inhibition of MMP-2. The range of concentrations of MMP-2i used was calculated to provide a level of MMP-2 inhibition comparable with that provided by TIMP-1 on the basis of their *Ki*. As illustrated in figure 7, the selective MMP-2i reduced neurite length after 24 h of exposure in a dose-dependent manner with a significant inhibitory effect (25%) detected at 100 nM, and a maximum effect (35%) at 1000 nM. The same concentrations of MMP-2i did not induce any changes in the surface of growth cones or in the number of actin rich protrusions (not shown).

## Discussion

The present work provides evidence that TIMP-1 modulates the morphology of neurons in a paracrine and autocrine manner. The paracrine actions of TIMP-1 appear to be mediated by its N-terminal domain which carries the inhibitory activity on MMPs, most likely on MMP-2. This proteinase, constitutively expressed by cortical neurons, is present in the cell soma, the neurites and the central domain of growth cones. Its inhibition by a selective MMP-2 inhibitor causes neurite length reductions comparable with those induced by TIMP-1, the latter equally affecting both dendrites and axons. The autocrine action of overexpressed TIMP-1/GFP causes an even more important reduction in neurite length and branching. Altogether, TIMP-1 emerges as a factor that modulates the size and number of actin rich structures (growth cones and microprocesses) and neurite outgrowth by inducing a reorganisation of the actin and tubulin cytoskeleton. Our findings may be of functional relevance mainly in post-lesion pre-scarring zones of the CNS where TIMP-1 is released at high levels by reactive astrocytes and by hyperactive neurons.

### TIMP-1 Affects the Morphology of Neurites, Growth Cones and F-Actin Rich Microprocesses

High expression of TIMP-1 in reactive astrocytes compared with much lower neuronal levels has been documented by different groups including ours [4]. In the present study only 3% of cells were identified as astrocytes by GFAP labelling, suggesting a minor contribution of these glial cells to TIMP-1 and MMP content. Although RT-PCR revealed the presence of TIMP-1 mRNA in cortical neurons, the protein was undetectable by western blot, in clear contrast with easily detected TIMP-1 (0.3–3 nM) in the supernatant of quiescent and LPS-stimulated astrocytes found in the present study (not shown) or in the cerebrospinal fluid of patients with neurodegenerative disorders [31]. The pathological concentrations of TIMP-1 in body fluids are within the range of those used in the present study that induced changes in the growth and morphology of neurons. The reduction of neurite length was concomitant with the increase in the number of small βIII-positive neurites (under 10 µm) and actin rich protrusions. These are highly dynamic structures that expand and retract constantly and it is possible that TIMP-1 stabilises them through an increase in their adhesion to extracellular substrates. An alternative, but non exclusive hypothesis is that TIMP-1 could act as a repellent cue for growth cones, subsequently leading to the emergence of lateral protrusions. Similar effects have been previously reported for repellent cues that inhibit growth cone motility of retinal ganglion cells [36], suggesting that secondary neurites emerging from the protrusions might compensate the sensory deficit of the leading growth cone. Although an increase in the size of growth cones is most commonly associated with expanding neurites, the 2-fold increase of the growth cone size observed upon TIMP-1 application is on the contrary concomitant with the reduction of neurite outgrowth. No clear explanation exists for such phenomenon, but it has been suggested that changes in microtubule dynamics may slow down neuritic outgrowth and facilitate the accumulation of material within the growth cone [37]. The question remains open as to whether TIMP-1 induces a transient collapse of growth cones in a way reminiscent of classical collapsing-like factors (ie, ephrin, semaphorins, etc). Investigating the kinetics of TIMP-1 action should provide further insight into the activation of signalling pathways preceding significant morphological changes. In this context, failure to detect changes in cofilin phosphorylation at 24 h after seeding does not preclude that such changes may actually occur at earlier time points.

### TIMP-1 Induced Changes in Neuronal Morphology Are Mediated by Its N-Terminal Domain

The finding that RXPO3R, a specific inhibitor of MMPs, essentially mimicked the effects of TIMP-1 provides indirect evidence that TIMP-1 targeting of MMPs is a causal event. Recent reports have suggested the implication of ADAMs in axon outgrowth and guiding in DRG and retinal ganglion cells [38]. The effects of the RXPO3R, which does not inhibit ADAMs [23], argues against a significant implication of these metalloproteinases as mediators of TIMP-1 effects on cortical neurons. A similar contingency would apply to MT-MMPs provided the poor inhibitory effect of TIMP-1 on the members of this MMP subfamily.

The most compelling evidence for a MMP inhibition-dependent action of TIMP-1 comes from data demonstrating that the truncated wild type N-terminal form of TIMP-1 reduces neurite outgrowth, whereas the inactive mutated form does not. Our data confirm in live cortical neurons previous studies showing that the truncated TIMP-1 retains metalloproteinase inhibitory activity in a cell-free system [39]. Altogether, these findings pinpoint the functional prevalence of the primary amino-acid sequence in the inhibitory properties of the N-terminal domain over post translational modifications such as glycosylation, not present in the truncated form produced by bacteria. From the above it follows that the C-terminal domain of TIMP-1 may be irrelevant in the molecular processes leading to the inhibition of neurite outgrowth.

### TIMP-1 Effects on Neurite Outgrowth of Cortical Neurons Appear Mediated in Part by the Inhibition of MMP-2

MMP-9 has been reported to contribute to axonal elongation in cerebellar neurons [17] but two findings in our study suggest that this proteinase does not play a critical role in neurite extension of cortical neurons; first, MMP-9 levels are very low as compared with MMP-2, and second, the N-terminal mutated form of TIMP-1 that selectively preserves MMP-9 inhibition had no effect on neurite outgrowth. Therefore, it is reasonable to conclude that the inhibitory effects of TIMP-1 on neurite outgrowth are not mediated by the inhibition of MMP-9. Instead, several lines of evidence point to MMP-2 as the most plausible target of TIMP-1 in cortical neurons. Indeed, unlike MMP-9, MMP-2 was constitutively expressed and particularly concentrated in the central domain of the growth cone, known to be enriched in microtubules [40], [41]. The exclusion of MMP-2 from areas rich in F-actin (ie, filopodia) suggests a possible functional connection of MMP-2 with the microtubular system, known to act as the principal structural component of neurite extension and retraction. In support of this hypothesis, we have recently shown that in neuronal cells, MMP-2-containing vesicles are transported along microtubules, in close interaction with the kinesin molecular motors [30]. The possibility that the effects of TIMP-1 result from the inhibition of MMP-2 finds further support in the observation that a synthetic selective MMP-2 inhibitor also reduces neurite length. Our findings, whether referred to the 24 or 48 h time-point reinforce recent data suggesting the involvement of MMP-2 in dendrite outgrowth of cortical neurons [19]. The same authors previously suggested that MMP-3 rather contributes to axonal growth [18]. In this context, we cannot exclude that the effects of mrTIMP-1 on axons, observed at 48 h, involve the inhibition of MMP-3.

The absence of MMP-2 from growth cone areas enriched in F-actin (ie, peripheral domain of the growth cone) is in line with the failure of selective MMP-2i to affect the size of growth cones or the number of actin rich microfilaments. These data suggest that TIMP-1 effects on actin rich structures involve the inhibition of another MMP likely to be inhibited by the broad spectrum MMP inhibitor RXPO3, which did increase the surface of growth cones. Altogether, these observations support a role for at least a TIMP-1/MMP-2 interaction in the control of neurite outgrowth in cortical neurons. This is in keeping with the role previously attributed to MMP-2 in the motility of DRG [20] and hippocampal [16] neurons, and astrocytes [42]. Our data also open the way to the investigation of TIMP-1 interactions that specifically support changes in the dynamics of the actin cytoskeleton.

### Overexpression of TIMP-1 in Cortical Neurons Reduces Neurite Outgrowth in an Autocrine Way

The important reduction of the neuritic arbour caused by transfected TIMP-1/GFP compared to GFP alone confirms that TIMP-1 interferes with normal outgrowth of cortical neurons regardless of its locus of synthesis. It is noteworthy that overexpression of TIMP-1/GFP causes a stronger reduction in neurite outgrowth than exogenous mrTIMP-1. These exacerbated effects may result from higher local concentrations of TIMP-1/GFP in and around the transfected neurons that inhibit MMP-2 more efficiently, but may also reflect the effects of the interactions of TIMP-1/GFP with other intracellular and pericellular targets that would be less available for the exogenously applied mrTIMP-1. Little is known about the interactions of TIMP-1 with non-metalloproteinase proteins. Recently, it has been suggested that in epithelial cells TIMP-1 interacts with the surface protein CD63 and modulates the tetraspanin/integrin β1 signaling complex [43]. Such interactions have not yet been reported for TIMP-1 in neurons, but the closely related TIMP-2 has been found to interact with α3β1 integrin in cultured cortical neurons and to promote neurite outgrowth and differentiation of PC12 cells through cell-cycle arrest [44]. Moreover, TIMP-3 promotes neuronal apoptosis by blocking the shedding of the tumour necrosis factor (TNF) superfamily of death receptors/ligands by MMP-3 [45], [46].

Overall, it appears that the different TIMPs have clearly distinct or even opposite effects in the nervous system, thus influencing cell survival and neurite outgrowth balance in developmental and/or post-lesion processes. Although the present study is focused on the effects of TIMP-1 on cultured primary neurons, our data may provide with clues to understand the functional consequences of TIMP-1 production in post-lesion or inflammatory pre-scarring zones of the CNS, but also to investigate the implication of TIMP-1 in learning processes [16], [47] and LTP [48] on the basis of its ability to shape neuronal and synaptic morphology. These challenging questions will deserve further attention in the future to ascertain the role of TIMP-1, and more generally of the MMP/TIMP system, in the CNS at the crossroads of physiology and pathology.

## Supporting Information

Figure S1 Inhibitory effect of mrTIMP on hrMMP-2 proteolytic activity. Fluorescence generated by cleavage of the fluorescein quenched substrate (Mcmat, 0.5 µg) is expressed as arbitrary fluorescence units (AFU). Mcmat cleavage by human recombinant MMP-2 (hr MMP-2, 20 ng) was inhibited by mouse recombinant TIMP-1 (mrTIMP-1, 100 ng) diluted in TCNB (Tris, CaCl2, NaCl, Brij-35 0.05%), reaching values equivalent to those representing the control (buffer and Mcmat, without proteinase). Note that the TCNB has no inhibitory effect on hrMMP-2 proteolytic activity.
(0.46 MB TIF)

Figure S2 Absence of axo-dendritic differentiation in cortical neurons 24 h after seeding. Fluorescent microphotographs showing immunolabelling for Tau-1 (green) and MAP2 (red) specific markers of axons and dendrites, respectively. Hoechst #33258 stained the nuclei (blue). At 24 h post-seeding, tau expression is low and essentially colocalised with MAP2, indicating no axonal differentiation at this stage. To the contrary, at 72 h post-seeding, specific labelling is shown for MAP2 in dendrites (arrowheads) and Tau-1 in axons (arrows). Scale bar 20 µm.
(1.73 MB TIF)

Figure S3 TIMP-1/GFP is not detected in the supernatant of transfected cortical neurons. Western blot from supernatants (S) and lysates (L) of N2a cells and neurons 24 or 48 h after transfection with TIMP-1/GFP constructs. The transgene is detected in N2a cells, where transfection rates are over 30%, whereas it is not detected in transfected neuronal cultures that yielded much lower transfection rates. Note that the molecular weight of the secreted transgene corresponds to the size of the fusion protein ∼60 kDa, while the lysates exhibit also a truncated form of the protein at ∼43 kDa. The western blot is representative of 3 independent experiments.
(0.33 MB TIF)

Figure S4 TIMP-1/GFP transfection and actin cytoskeleton labelling. Fluorescent microphtographs showing anti-GFP immunostaining (green) and F-actin phallodin labelling (red). Hoechst #33258 stained the nuclei (blue). Note that transfected neurons appear well integrated in the neuronal circuitry and most neurites and growth cones are intermingled with neuritic extensions from non-transfected neurons. Scale bars 20 µm.
(3.81 MB TIF)
