# Supplementary figures and images for: A New Role for TIMP-1 in Modulating Neurite Outgrowth and Morphology of Cortical Neurons
Source: PLoS One. 2009 Dec 14;4(12):e8289. doi: 10.1371/journal.pone.0008289 (PMC2788270; doi:10.1371/journal.pone.0008289)

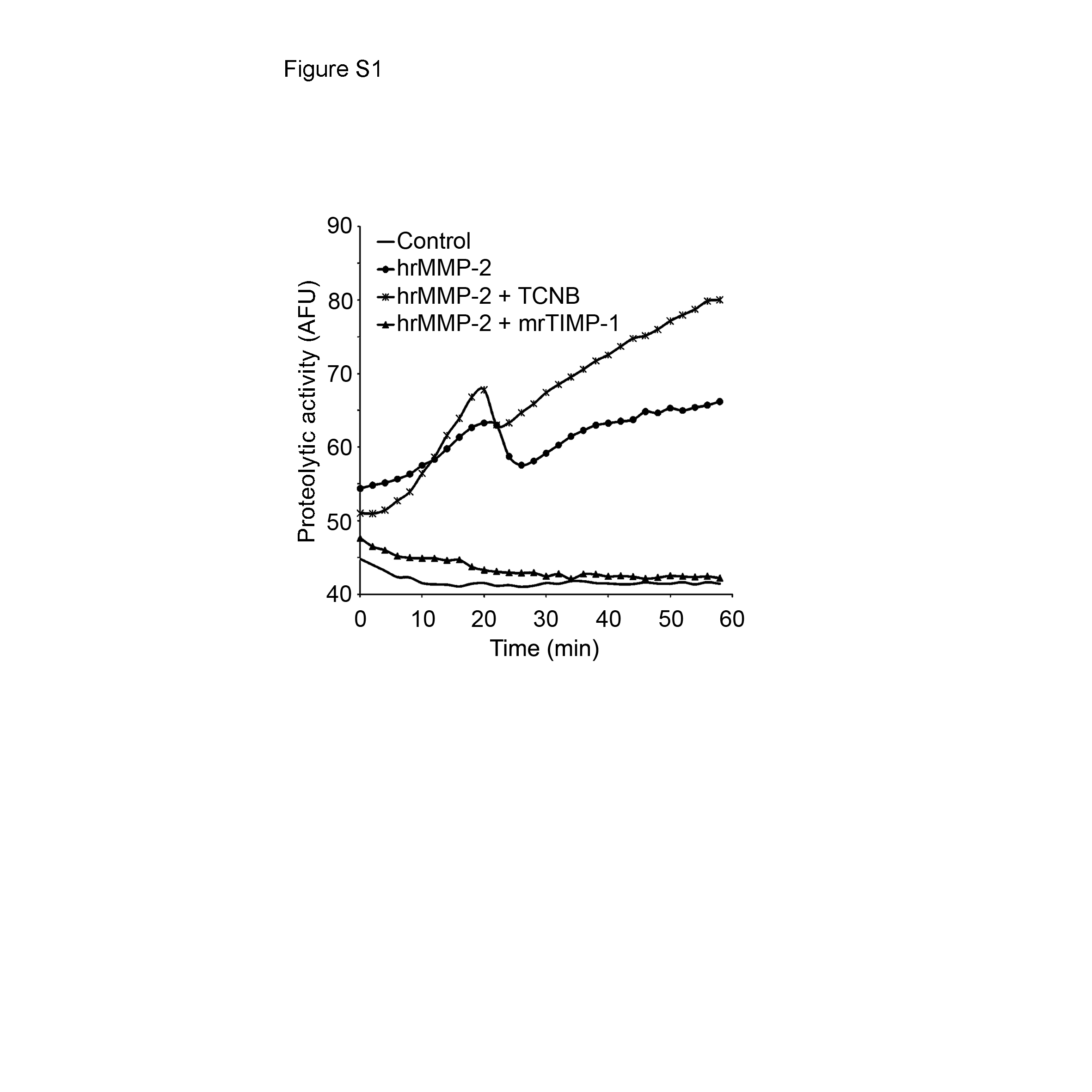

Supplement: Figure S1 — Inhibitory effect of mrTIMP on hrMMP-2 proteolytic activity. Fluorescence generated by cleavage of the fluorescein quenched substrate (Mcmat, 0.5 µg) is expressed as arbitrary fluorescence units (AFU). Mcmat cleavage by human recombinant MMP-2 (hr MMP-2, 20 ng) was inhibited by mouse recombinant TIMP-1 (mrTIMP-1, 100 ng) diluted in TCNB (Tris, CaCl2, NaCl, Brij-35 0.05%), reaching values equivalent to those representing the control (buffer and Mcmat, without proteinase). Note that the TCNB has no inhibitory effect on hrMMP-2 proteolytic activity. (0.46 MB TIF) [file pone.0008289.s001.tif]

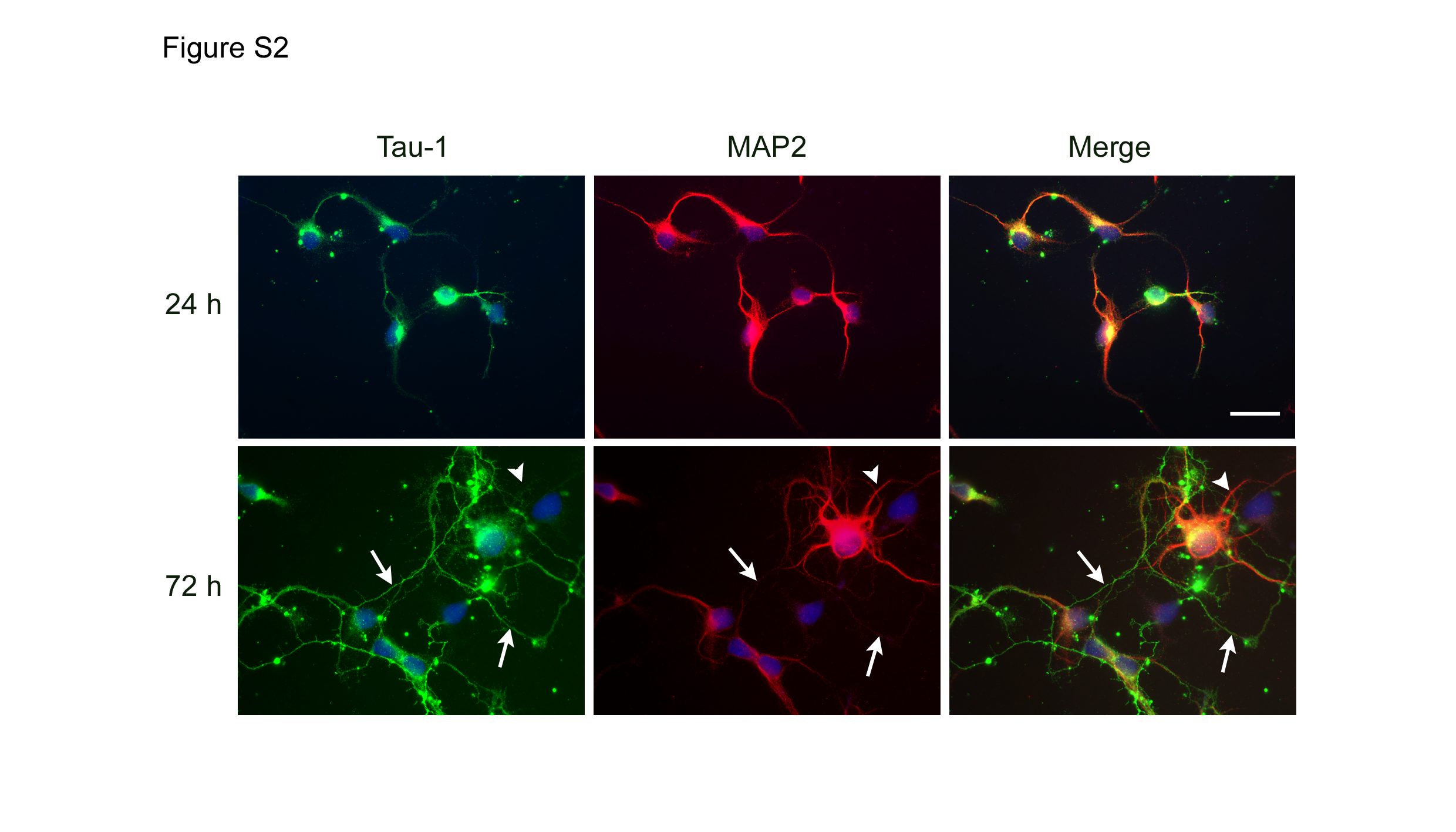

Supplement: Figure S2 — Absence of axo-dendritic differentiation in cortical neurons 24 h after seeding. Fluorescent microphotographs showing immunolabelling for Tau-1 (green) and MAP2 (red) specific markers of axons and dendrites, respectively. Hoechst #33258 stained the nuclei (blue). At 24 h post-seeding, tau expression is low and essentially colocalised with MAP2, indicating no axonal differentiation at this stage. To the contrary, at 72 h post-seeding, specific labelling is shown for MAP2 in dendrites (arrowheads) and Tau-1 in axons (arrows). Scale bar 20 µm. (1.73 MB TIF) [file pone.0008289.s002.tif]

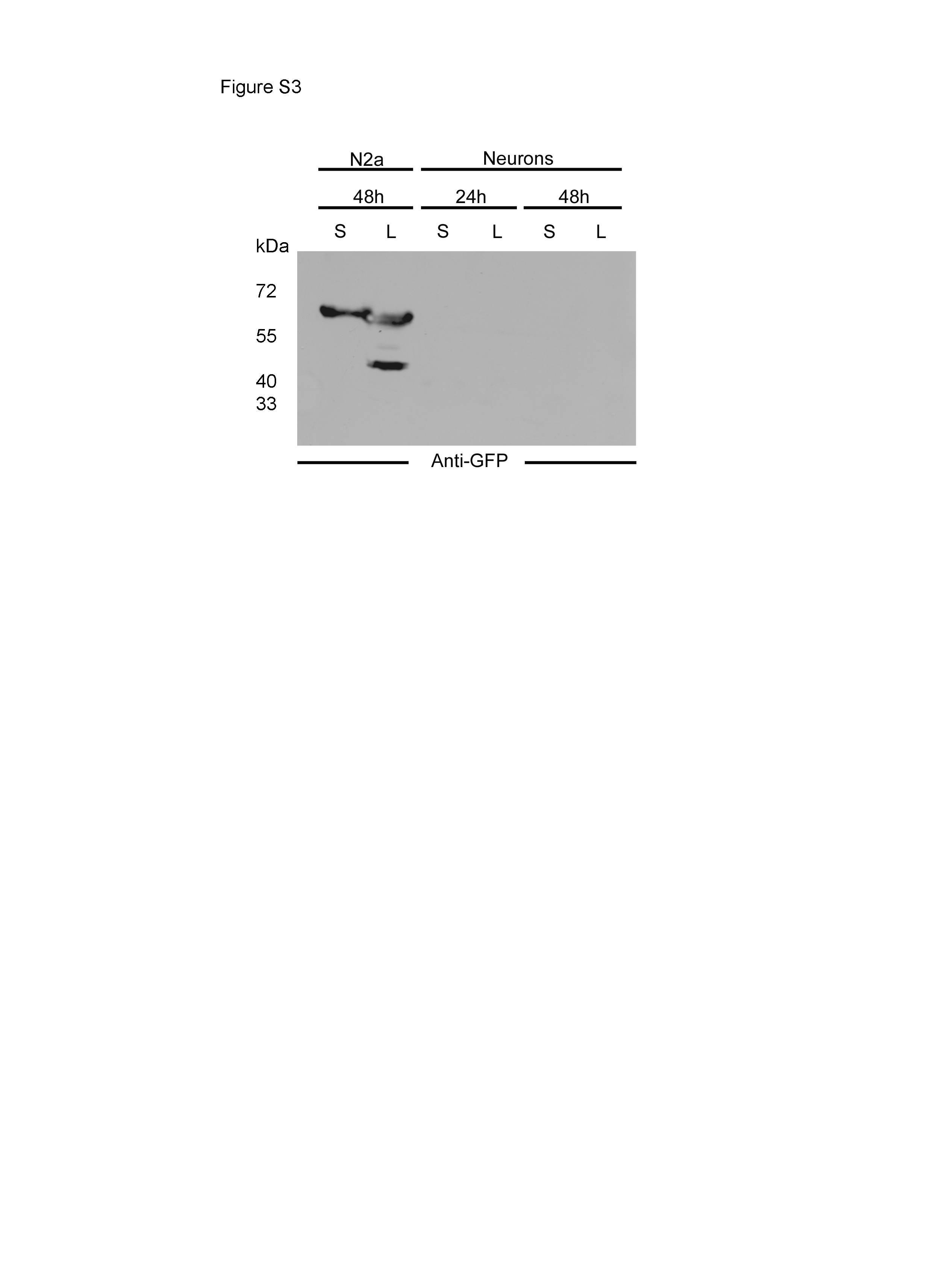

Supplement: Figure S3 — TIMP-1/GFP is not detected in the supernatant of transfected cortical neurons. Western blot from supernatants (S) and lysates (L) of N2a cells and neurons 24 or 48 h after transfection with TIMP-1/GFP constructs. The transgene is detected in N2a cells, where transfection rates are over 30%, whereas it is not detected in transfected neuronal cultures that yielded much lower transfection rates. Note that the molecular weight of the secreted transgene corresponds to the size of the fusion protein ∼60 kDa, while the lysates exhibit also a truncated form of the protein at ∼43 kDa. The western blot is representative of 3 independent experiments. (0.33 MB TIF) [file pone.0008289.s003.tif]

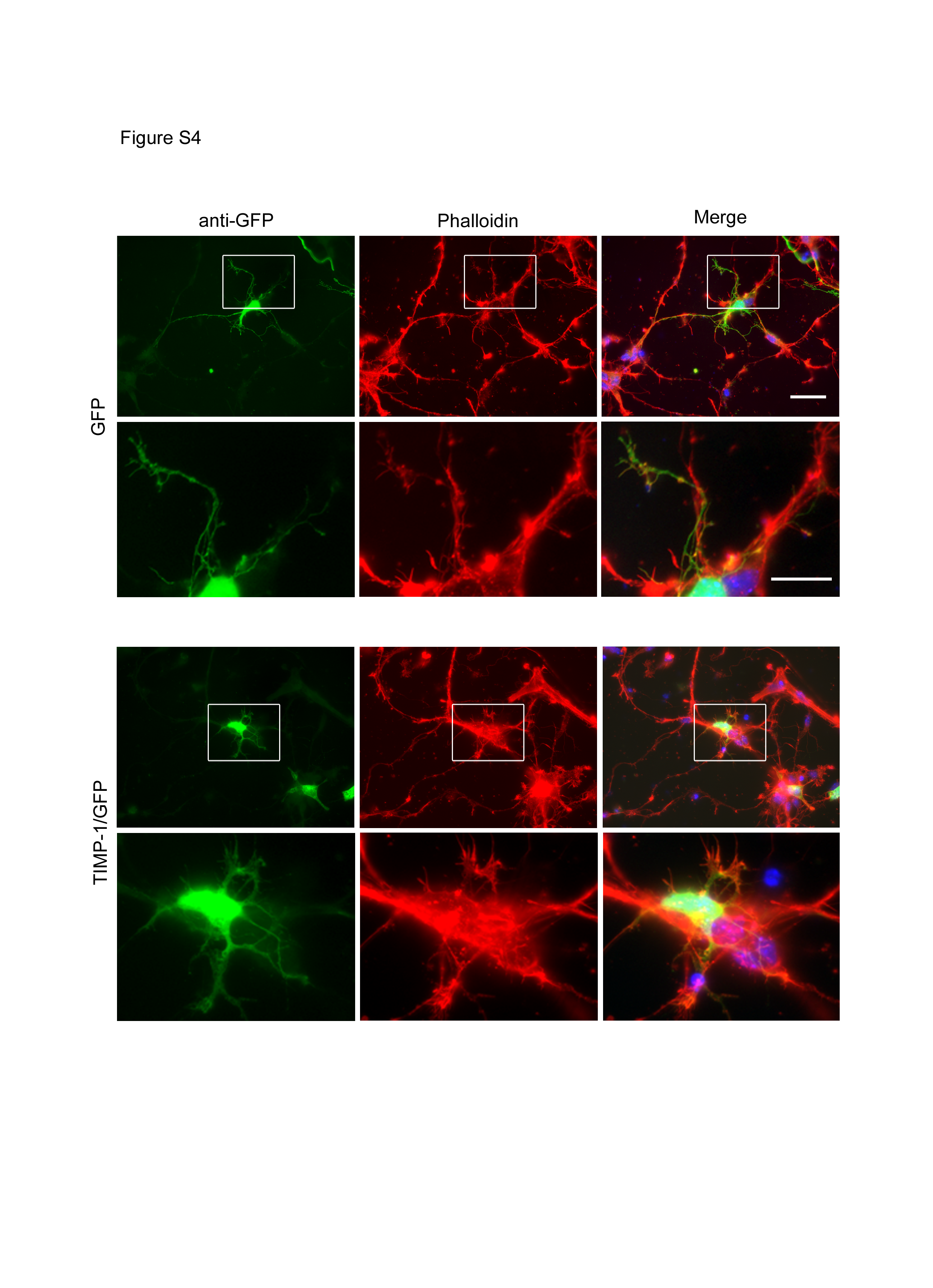

Supplement: Figure S4 — TIMP-1/GFP transfection and actin cytoskeleton labelling. Fluorescent microphtographs showing anti-GFP immunostaining (green) and F-actin phallodin labelling (red). Hoechst #33258 stained the nuclei (blue). Note that transfected neurons appear well integrated in the neuronal circuitry and most neurites and growth cones are intermingled with neuritic extensions from non-transfected neurons. Scale bars 20 µm. (3.81 MB TIF) [file pone.0008289.s004.tif]
